# *Streptomyces griseorubens* as a microbial cell factory for extracellular uricase production and bioprocess optimization using statistical approach

**DOI:** 10.1186/s12934-024-02561-4

**Published:** 2024-11-12

**Authors:** Noura El-Ahmady El-Naggar, Sara M. El-Ewasy, Nancy M. El-Shweihy

**Affiliations:** Department of Bioprocess Development, Genetic Engineering and Biotechnology Research Institute, City of Scientific Research and Technological Applications (SRTA-City), New Borg El- Arab City, 21934 Alexandria Egypt

**Keywords:** *Streptomyces*, Uricase production, Identification, 16S rDNA sequencing, SEM, Optimization, Plackett–Burman design, CCD

## Abstract

**Background:**

Uricase is a bio-drug used to reduce urate accumulation in gout disease. Thus, there is a continuous demand for screening soil samples derived from a variety of different sources in order to isolate a strain that possesses a high potential for producing uricase.

**Methods:**

*Streptomyces* sp. strain NEAE-5 demonstrated a significant capacity for uricase production was identified based on the physiological, morphological and biochemical characteristics, as well as 16S rDNA sequencing analysis. Using a Plackett–Burman statistical design, the impact of eighteen process factors on uricase production by *Streptomyces griseorubens* strain NEAE-5 was investigated. Using central composite design, the most important variables that had a favourable positive impact on uricase production by *Streptomyces griseorubens* strain NEAE-5 were further optimized.

**Results:**

It is clear that the morphological and chemotaxonomic features of *Streptomyces* sp. strain NEAE-5 are typical for the *Streptomyces* genus. Phylogenetic analysis indicated that *Streptomyces* sp. strain NEAE-5 belongs to the genus *Streptomyces* and closely related to* Streptomyces griseorubens* which it has a 95–96% identity in 16S rDNA gene sequencing. Accordingly, the strain is proposed to be identified as *Streptomyces griseorubens* strain NEAE-5. The three factors that had the significant positive impacts on uricase production were uric acid, hypoxanthine, and yeast extract. As a result, the best conditions for achieving the highest experimental uricase production by *Streptomyces griseorubens* strain NEAE-5 after central composite design were (g/L): uric acid 6.96, glycerol 5, hypoxanthine 5.51, MgSO_4_.7H_2_O 0.1, KNO_3_ 2, CaCl_2_ 0.5, K_2_HPO_4_ 0.5, NaCl 0.5, yeast extract 1.08. In addition, the period of incubation is seven days, pH 7.5 and 37 °C with an inoculum size of 2 mL (10^5^ cfu/mL) /100 mL medium.

**Conclusions:**

After optimization, the obtained uricase activity was 120.35 U/mL, indicating that the *Streptomyces griseorubens* strain NEAE-5 is a potent uricase producer and that the statistical approach used for optimization was appropriate.

## Introduction

Gout is a painful metabolic abnormality disease caused by the accumulation and precipitation of uric acid crystals in the bloodstream (hyperuricemia). It may also result in the formation of uric acid crystals in the kidneys, which form stones [1]. Overproduction of uric acid can lead to Lesch-Nyhan syndrome; chronic kidney failure and some organic acidemias [2]. Uricase is an oxidoreductase enzyme that transforms uric acid into carbon dioxide, hydrogen peroxide, and allantoin. The kidneys quickly excrete allantoin because it is more soluble and less toxic [3]. Medicinally; uricase is utilized as a therapeutic protein to reduce high urate levels, treat hyperuricemia and gout [4, 5]. Uricase is a potent, safe, and fast-acting urate-lowering drug used to treat and prevent hyperuricemia induced by organ transplantation and tumor lysis [6–8]. In the cosmetics industry, uricase is added to the formulas of hair dyes [9]. In clinical laboratories, uricase is frequently used as a diagnostic tool to determine the amount of uric acid present in blood serum and other biological fluids. This is accomplished through coupling of uricase with a 4-aminoantipyrine-peroxidase system to form a quinoneimine dye [10–12].

Bacteria such as *Pseudomonas putida* ([13], *Gliocladium viride* [14],* Bacillus subtilis* [15], *Pseudomonas aeruginosa* [16],* Microbacterium* sp. ZZJ4-1 [17], *Gliomastix gueg* [18] and *Nocardi farcinica* [19] were used for the production of uricase. Some fungi were also capable of producing uricase such as* Aspergillus welwitschiae* strain 1–4 [4], *Aspergillus terreus* as documented by Tohamy and Shindia [20], *Rhizopus oryzae* as reported by Farley and Santosa [21] and* Mucor hiemalis* [22], *Aspergillus niger* [23] and yeasts as* Candida utilis* [24].

Despite the fact that uricase has been produced from numerous sources, including genetically engineered microbes, novel microbial producers of uricase are being explored in an effort to produce the enzyme with improved yield [22]. *Streptomyces* is the largest and most significant genus in the actinomycetales order. Up to 90 percent of actinomycetes identified from soil samples belong to the *Streptomyces* genus [25]. *Streptomyces* is a prolific source of diverse and valuable natural bioactive compounds including enzymes, antibiotics, nanoparticles etc. [26–30]. *Streptomyces* such as *Streptomyces rochei* NEAE–25 has been used to produce uricase [31].

The optimization of nutritional and environmental variables that significantly affect the microbial uricase production is crucial because it could affect downstream product separation costs and product concentration levels [32]. Traditionally, one factor at a time optimization has involved changing one factor while keeping all other factors constant. This approach ignores the interactions between different parameters, and it is both expensive and time-consuming [33]. Therefore, statistical optimization techniques have been developed. Response surface methodology (RSM) is an effective technique for predicting the optimal conditions while reducing the number of trials needed. RSM is also helpful in determining the interactions between the examined parameters and the response within the tested range [34].

The aim of this investigation was to optimize the process factors, to improve the production of uricase by the local isolate *Streptomyces* sp. strain NEAE-5 and to identify the selected strain.

To the best of our knowledge, uricase production by *Streptomyces* has only been reported from a limited number of species. This paper is the first to study the uricase production by *Streptomyces griseorubens.*

## Materials and methods

### Microorganism and culture maintenance

In this investigation, the first author isolated *Streptomyces* sp. strain NEAE-5 from a soil sample taken from the northwest coast of Egypt at Borg El-Arab City. The strain was isolated on Petri dishes supplied with the starch nitrate agar medium of the following composition (g/L): KNO_3_: 2, starch: 20, MgSO_4_ 0.7H_2_O: 0.5, K_2_HPO_4_:1, FeSO_4_.7H_2_O: 0.01, NaCl: 0.5, CaCO_3_: 3, agar: 20 and distilled water up to 1 L. After incubation at 30 °C for 7 days, the plates were stored at 4 °C.The isolate was stored as a spore suspension in glycerol at a concentration of 20% (v/v) and a temperature of -20 degrees Celsius.

### The uricase-producing capacity of *Streptomyces* sp. strain NEAE-5

In our previous study [31], the total of one hundred and thirty morphologically different actinomycete strains. All these isolates were purified and their capabilities to produce uricase were assessed using the conventional spot inoculation method on plates containing uric acid induction medium. The medium ‘s composition in (g/L): glycerol: 30, uric acid: 5, K_2_HPO_4_:1, NaCl: 5; CaCl_2_: 0.1, MgSO_4_ 0.7H_2_O: 0.2, agar: 20, and distilled water up to 1 L. pH was adjusted to 6.8 [31]. After seven days incubation at 30 °C, formation of clear zones surrounding the colonies indicated the uricase production capability. The strain's uricase production capability was further verified under submerged fermentation conditions. Among the most promising isolates, *Streptomyces* sp. strain NEAE-5 was selected for further studies.

### Submerged-fermentation

The inoculum was prepared by dispensing 50 mL of liquid uric acid fermentation medium into 250 mL Erlenmeyer flasks, which were then sterilized and inoculated with three 9-mm discs of *Streptomyces* sp. strain NEAE-5 obtained from a 7-day-old starch nitrate agar culture plate started from a single colony. The flasks were subsequently incubated in a shaker incubator at 200 rpm and 30 °C for two days, after which the resulting inoculum was utilized in subsequent experiments.

In the submerged-fermentation experiments, 250 mL Erlenmeyer flasks were filled with 50 mL of liquid uric acid fermentation medium, sanitized, and inoculated with 2% (v/v) of the previously prepared inoculum. The inoculated flasks were then incubated in a shaker incubator at 30 °C for 4 to 7 days, as specified by the experimental trials. The mycelia of *Streptomyces* sp. strain NEAE-5 were centrifuged for 15 min. at 5000 × *g* following the incubation period. The uricase activity in the resulting cell-free supernatant was measured [31].

### Uricase assay

The uricase activity was quantified following the protocol established by El-Naggar [31]. Uricase activity was evaluated by incubating 300 μL enzyme solution with a combination of 400 μL sodium borate buffer (pH 8.5, 0.1 M) containing 2 mM uric acid, 100 μL phenol (1.5%), 50 μL peroxidase (15 U/mL) and 150 μL 4-aminoantipyrine (30 mM) at 37 ºC for 30 min. To stop the reaction, 200 μL of 0.1 M potassium cyanide solution was added. In the blank, the reaction mixture was mixed with a potassium cyanide solution added prior to the addition of crude enzyme. A spectrophotometer was used to measure the absorbance against to the blank at a wavelength of 540 nm. One unit of uricase was defined as the quantity of enzyme that generates one micromole of hydrogen peroxide per minute under the assay conditions.

### Morphological and cultural characteristics

Following the method described by El-Naggar et al. [28], the spore surface ornamentation and spore chain morphology of *Streptomyces* sp. strain NEAE-5 were examined using SEM (scanning electron microscopy). Cultural characteristics, including formation of diffusible pigments, substrate mycelial pigmentation and aerial spore-mass color were studied following the procedures described by Shirling and Gottlieb [35] on Petri plates containing ISP medium which are inorganic salt starch agar; yeast extract-malt extract agar; peptone-yeast extract iron agar; oatmeal agar glycerol- asparagine agar; tryptone-yeast extract agar or tyrosine agar. At 30 °C, Petri dishes were incubated for 14 days.

### Physiological characteristics

The utilization of different carbon sources by the strain was assessed on the basal ISP medium 9 plates, and the production of melanin pigment was examined on ISP media 1, 6, and 7 using the procedures described by Shirling and Gottlieb [35]. Protease production, gelatin liquefaction, lecithinase activity, the reduction of nitrates to nitrites, growth in the existence of sodium chloride and sodium chloride tolerance and the capacity for producing α-amylase, its tendency to peptonize or coagulate milk, and the strain's ability to produce cellulase and decompose cellulose were determined using the procedures described by El-Naggar and Abdelwahed [26]. The ability of *Streptomyces* sp. strain NEAE-5 to inhibit the proliferation of *Aspergillus niger*, *Pseudomonas fluorescens*,* Pseudomonas aeruginosa*, *Escherichia coli*, *Bacillus subtilis*,* Saccharomyces cerevisiae*, *Candida albicans* was discovered using the procedures described by El-Naggar and Hamouda [29]. Based on Bergey’s Manual of Systematic Bacteriology-volume five of the actinobacteria, it is very important to perform all physiological, morphological, and cultural characteristics in addition to 16S rDNA sequence analysis to fully identify the actinobacteria to the species level. The identification of actinobacteria to the species level based mainly on the physiological [36].

### 16S rDNA sequencing, sequence alignment and phylogenetic analysis

InstaGene Matrix (Bio-Rad, USA) was used to prepare the genomic DNA template in accordance with the manufacturer's instructions, the supernatant used for PCR. The PCR amplification reaction was conducted, and the resulting product was analyzed by agarose gel electrophoresis and purified using the protocol described by El-Naggar et al. [37]. The PCR amplification reaction was performed in a total volume of 100 μL, which contained 0.5 μL Taq polymerase, 1 μL DNA (50 ng), 10 μL PCR buffer, 3.5 μL 25 mM MgCl_2_ and 10 μL of 250 mM deoxyribonucleotide 5’-triphosphate (dNTP’s), 4 μL of 10 pmol (each) the universal 16S rRNA primer pair of 27 F (5'-AGAGTTTGATCMTGCCTCAG-3') and 1492 R (5'-TACGGYTACCTTGTTACGACTT-3') and water was added up to 100 μL. The PCR- thermocycler (Peltier ermal Cycler PTC-225, Macrogen, Korea) was programmed as follows: 5 min denaturation at 94°C, followed by 35 amplification cycles of 1 min at 94°C, 1 min of annealing at 55°C, and 2 min of extension at 72°C, followed by a 10 min final extension at 72°C. Montage PCR Clean up kit (Millipore) was used to remove unincorporated PCR primers and dNTPs from PCR products. The purified PCR product was two-directional sequenced by using 2 primers (27 F and 1492 R). 16S rDNA sequencing was performed by Macrogen Company, Seoul, Korea (http://www.macrogen.com) using Big Dye terminator cycle sequencing kit (Applied BioSystems, USA). Sequencing products were resolved on an Applied Biosystems model 3730XL automated DNA sequencing system (Applied BioSystems, USA). The 16S rDNA gene sequence was aligned with sequences obtained from different databases using the BLAST program (https://blast.ncbi.nlm.nih.gov/Blast.cgi?PAGE_TYPE=BlastSearch) [38]. The MEGA-X software package was used in order to conduct the phylogenetic analysis [39].

### Statistical optimization of uricase production using Plackett–Burman design

Uricase production from an actinomycete strain is mostly dependent on various factors such as uric acid concentrations, temperature, pH, carbon source, and nitrogen source. Consequently, these factors must be optimized to increase uricase yields. The statistical approach known as “ Plackett–Burman design” commonly used for screening and identification of significant process conditions for maximizing enzyme production [33, 40]. The process factors affecting uricase production by *Streptomyces griseorubens* were selected based on data collected from previous studies, including our own [31] and investigated using a Plackett–Burman design. Eighteen assigned factors including L-asparagine, glutamic acid, allantoin, hypoxanthine, pH, incubation time, temperature, inoculum size, glycerol, glucose, yeast extract, KNO_3_, K_2_HPO_4_ NaCl, MgSO_4_.7H_2_O, and CaCl_2_, uric acid and peptone were tested for their impact on uricase production. All trials were conducted in duplicate. A dummy variable (unassigned variable; D_1_) was used to estimate experimental errors. The uricase activity was measured as the response.

The data obtained from Plackett–Burman experiments were fitted to a first-order equation:

1 $$Y = \,\beta _{0} \, + \,\sum {\beta _{i} } X_{i}$$

where Y, *β*_0_, *β*_i_, and X_i_ represent the predicted uricase activity, the model`s intercept, linear coefficient, and levels of the independent variables; respectively.

### Central composite design (CCD)

The results of the Plackett–Burman experiments revealed that uric acid, hypoxanthine, and yeast extract concentrations had the highest contribution and significant impact on uricase production. Therefore, the central composite design was used to further optimize three factors and to improve uricase production. These factors (coded as X_1_, X_2_ and X_3_) were studied at five distinct levels (+ 1.68, + 1, 0, − 1, − 1.68). A total of 20 experiments were conducted, each using a different combination of the three factors. The data obtained from these experiments were fitted to a second-order polynomial equation found below:

2 $$Y = \beta_{0} + \sum\limits_{i} {\beta_{i} X_{i} + \sum\limits_{ii} {\beta_{ii} X_{i}^{2} } } + \sum\limits_{ij} {\beta_{ij} X_{i} X_{j} }$$

where Y, *β*_0_, *β*_i_, *β*_ii_, *β*_ij_, represent the predicted uricase activity, the regression coefficients, the linear, quadratic, interaction coefficients; respectively. X_i_ represent the coded levels of the factors. Each experiment was performed in triplicates and the average was taken as the response variable.

### Statistical analysis

To analyze the collected data from the optimization experiments, a multiple regression analysis was performed using Design Expert for Windows version 12 (Stat-Ease Inc., USA). Three-dimensional surface plots were created with the help of the statistical programme STATISTICA (StatSoft Inc., Tulsa, USA, Version 8.0).

## Results and discussion

### Potentiality of *Streptomyces griseorubens* for uricase production

**Fig. 1:**
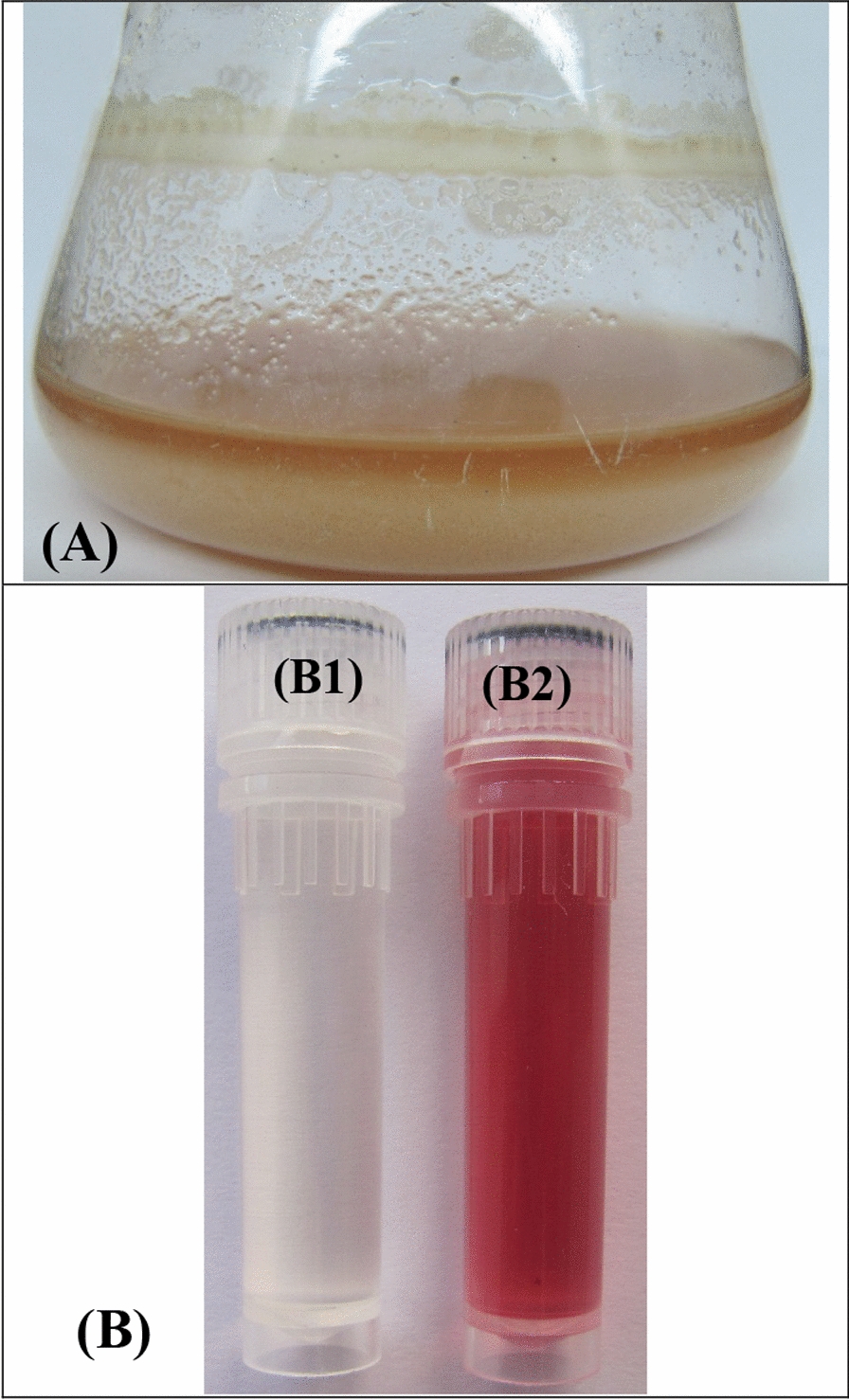
**A** Production of uricase by *Streptomyces griseorubens* under submerged fermentation conditions to confirm uricase production and** B** uricase assay reaction color; B1 is the control tube while B2 is the test tube

The plate method was used to determine whether or not *Streptomyces* sp. strain NEAE-5 has the capacity to produce uricase. *Streptomyces* sp. strain NEAE-5 has a distinct clear zone surrounding its growth (about 4 cm in diameter), which suggests that it can produce uricase. Quantification of uricase activity was carried out under submerged fermentation conditions, as depicted in Fig. 1A, and it was found that under submerged fermentation conditions, *Streptomyces* sp. strain NEAE-5 produced 30.45 U/mL. The uricase assay reaction color is illustrated in Fig. 1B.

### Morphological and cultural characteristics

**Fig. 2:**
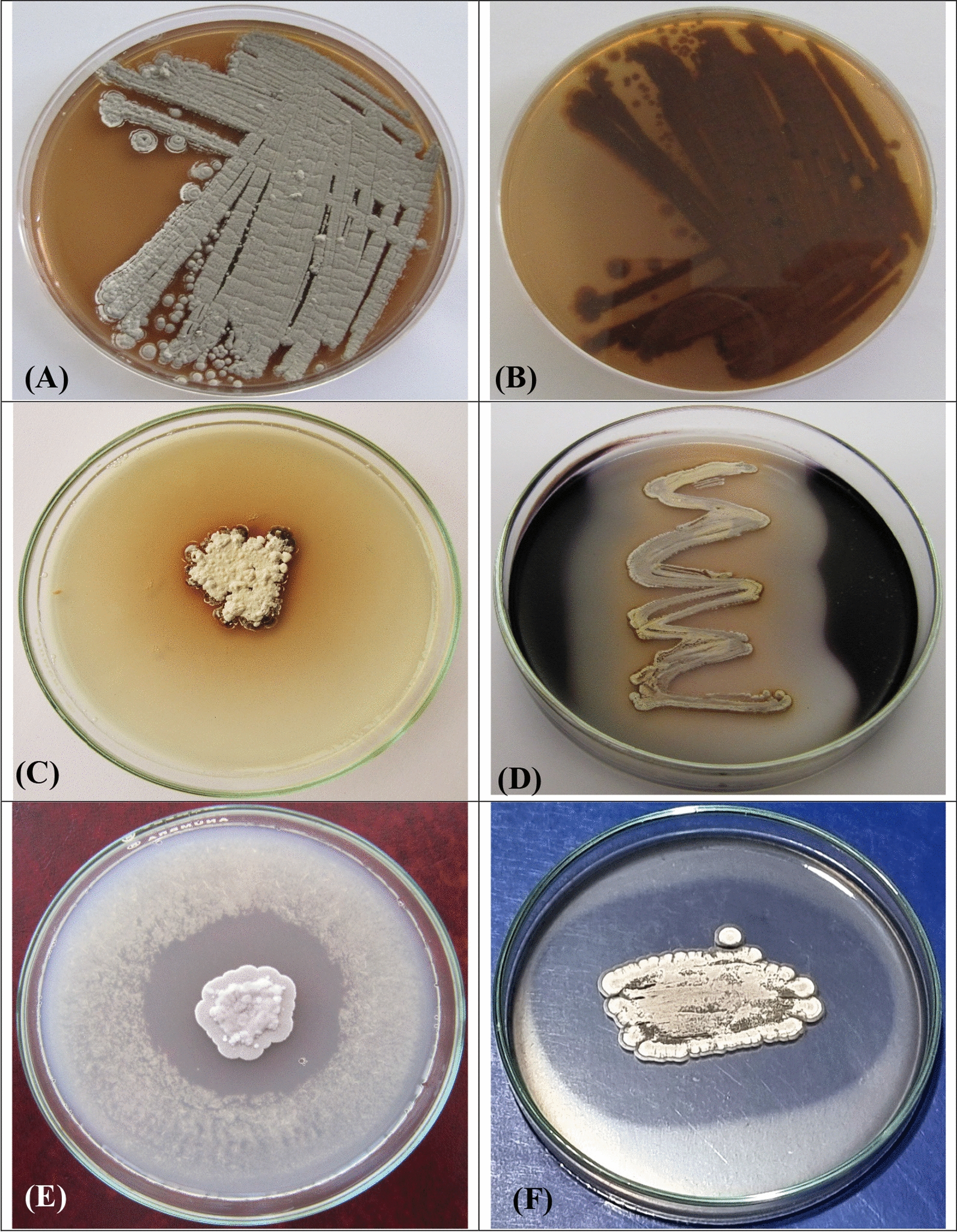
**A** reflect the color of aerial mycelium, **B** reflect the color of substrate mycelium **C** Plate assay showing the melanoid pigment production, **D** zone of hydrolysis of starch, **E** plate screening for the uricase activity and **F** protease production by *Streptomyces* sp. NEAE-5

Table 1 summarizes the growth as well as the cultural features of *Streptomyces* sp. strain NEAE-5 on different ISP media. This strain exhibited robust growth on ISP media 1–5 and 7. When grown on yeast extract-malt extract agar medium, the colour of the aerial mass is gray (Fig. 2A), whereas its substrate mycelium color is brown (Fig. 2B). The strain exhibited no fragmentation of mycelium, and verticils were not observed. Brown dispersible pigments were observed on most test media.

**Table 1:** Culture properties of strain *Streptomyces* sp. NEAE-5

Medium	Color of		
	Aerial mycelium	Substrate mycelium	Diffusible pigment
Tryptone-yeast extract agar (ISP medium 1)	Grey	Brown	Brown
ISP 2 medium (Yeast extract -malt extract agar)	Grey	Brown	Brown
ISP 3 medium (Oatmeal agar)	Grey	Faint brown	Non-pigmented
ISP 4 medium (Inorganic salt-starch agar)	Greyish brown	Brown	Brown
ISP 5 medium (Glycerol asparagine agar)	Brownish grey	Faint brown	Faint brown
ISP 6 medium (Peptone-yeast extract iron agar)	Brownish grey	Faint brown	Faint brown
ISP 7 medium (Tyrosine agar)	Beige	Brown	Brown

### Physiological characteristics

Regarding its chemotaxonomic characteristics, the optimal growth temperature and pH range of *Streptomyces* sp. strain NEAE-5 were determined to be 30–37 °C and 6.0–7.5, respectively. On most test media, the diffusible pigments appeared brown. Table 2 presents the positive reactions of *Streptomyces* sp. strain NEAE-5, such as milk peptonization and coagulation, melanin production (Fig. 2C), gelatin liquification, the hydrolysis of starch (Fig. 2D), and the reduction of nitrate to nitrite. The strain also exhibited positive reactions for the production of α–amylase, cellulase, uricase (Fig. 2E), protease (Fig. 2F), gelatinase, and asparaginase. among others, but a negative reaction for lecithin degradation. *Streptomyces* sp. strain NEAE-5 was unable to inhibit the growth of numerous microorganisms including *Aspergillus niger*,* Saccharomyces cerevisiae*,* Candida albicans*, *Pseudomonas fluorescens*,* Pseudomonas aeruginosa*,* Bacillus subtilis* and *Escherichia coli*. The strain utilized various sugars during its growth, including D ( +) mannose, D ( +) xylose, maltose, lactose, D ( +) glucose, D ( +) galactose, D (–) fructose, cellulose, and sucrose, while ribose was not utilized. *Streptomyces* sp. strain NEAE-5 was assigned to the genus *Streptomyces* based on its physiological, morphological, cultural, and chemotaxonomic characteristics [41].

**Table 2:** Characteristics of *Streptomyces* sp. NEAE -5

Characteristics	*Streptomyces* sp. NEAE -5
Production of diffusible pigment	Yellowish brown
Substrate mycelium on ISP medium 2	Yellowish brown
Aerial mycelium on ISP medium 2	Gray
Spore chain morphology	Rectiflexibilies
Spore shape	Ovoid
Spore surface	Spiny
Lecithinase activity	–
Protease production	+
L-asparaginase	+
Cellulose decomposition	+
Gelatin liquefaction	+
Peptonization of milk	+
Coagulation of milk	+
Starch hydrolysis	+
Melanoid pigment	+
Reduction of nitrates to nitrite	+
NaCl tolerance	9%
Growth on sole carbon sources (1.0%, w/v)	
D ( +) Galactose	+
D ( +) Glucose	+
Maltose	+
α-Lactose	+
Sucrose	+
D ( +) Xylose	+
D ( +) Xylose	+
D ( +) Mannose	+
D (–) Fructose	+
D (−) Fructose	+
Ribose	–
Cellulose	+
Antagonistic activity	
*Aspergillus niger*	–
*Sacchromyces cerevisiae*	–
*Pseudomonas fluorescens*	–
*Candida albicans*	–
*Bacillus subtilis*	–
*Pseudomonas aeruginosa*	–
*Escherichia coli*	–

#### Scanning electron microscopy (SEM) of *Streptomyces* sp.

**Fig. 3:**
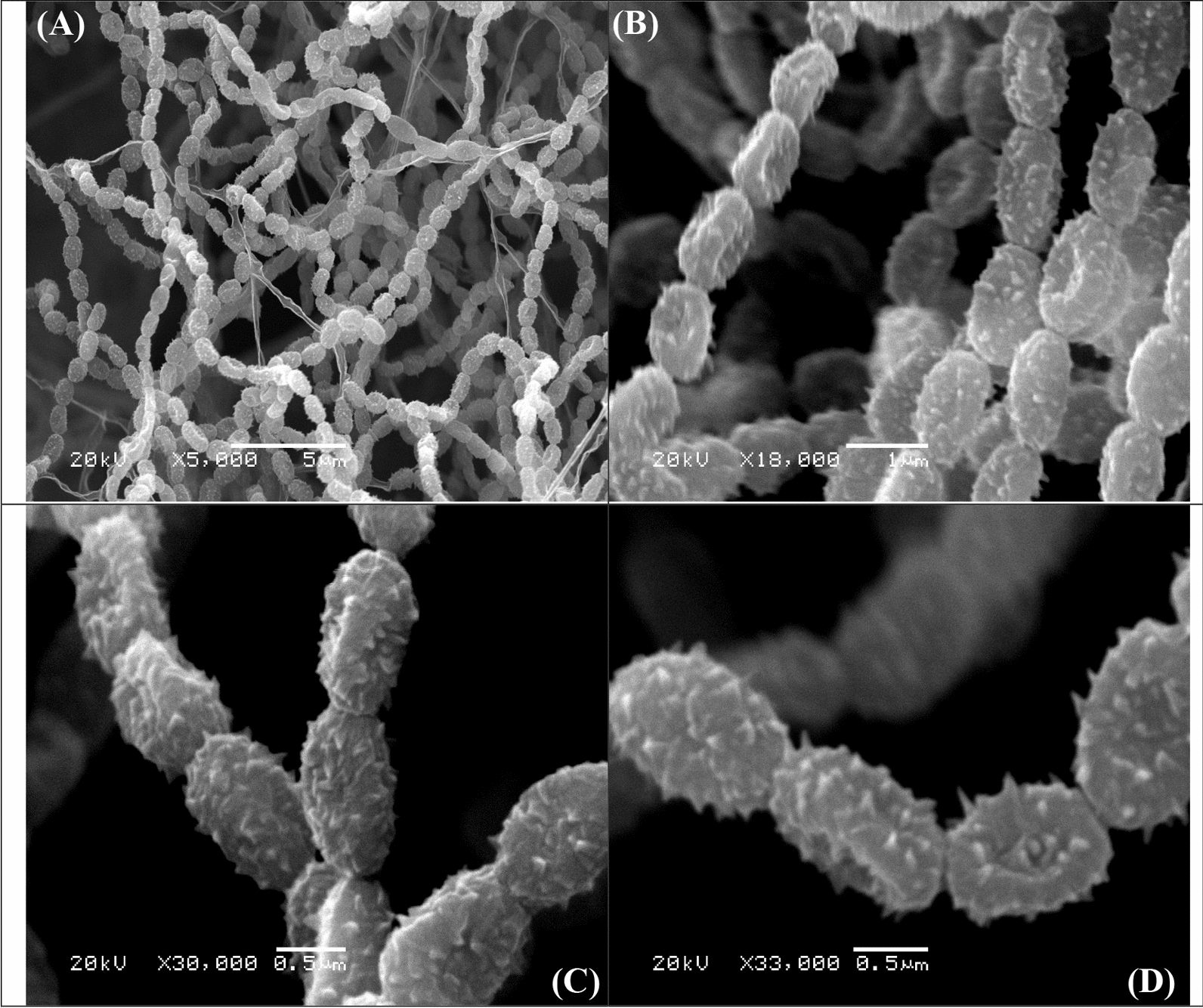
Scanning electron micrographs of *Streptomyces* sp. showing variations in spore chain morphology; **A**–**D** at different magnifications 5000x, 18000x, 30,000 and 33000x; respectively

The scanning electron microscopy was used to examine the spore surface ornamentation and spore chain morphology of *Streptomyces* sp. strain NEAE-5 The obtained SEM images revealed long, straight spore-chains of *Rectiflexibiles* type, comprising over 50 ovoid, spiny-surfaced spores (as shown in Fig. 3A–D).

### Phylogenetic analysis

**Fig. 4:**
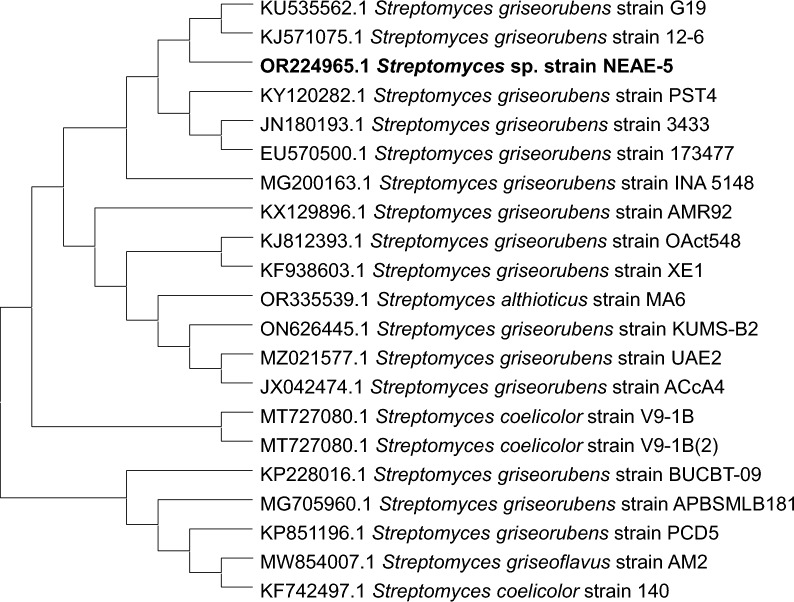
Neighbor-joining phylogenetic tree. MEGA-X was used to carry out evolutionary analyses. The evolutionary distances were determined using the Tamura-Nei algorithm and are expressed in terms of the number of base substitutions per site. All ambiguous locations were eliminated for each pair of sequences (pairwise deletion option)

The BLAST programme [38] was utilized in order to align the 16S rDNA gene sequence of *Streptomyces* sp. strain NEAE-5 with the 16S rDNA gene sequences of other *Streptomyces* species that were found in various databases. The sequence was submitted to GenBank, where it was assigned the accession number OR224965.1. The results of the neighbor-joining phylogenetic tree analysis [42] showed that the *Streptomyces* sp. strain NEAE-5 has the closest relationship to *Streptomyces griseorubens* strain G19 (accession number: KU535562.1, 16S rDNA gene sequence similarity of 95.51%) and *Streptomyces griseorubens* strain 12–6 (accession number: KJ571075.1, 16S rDNA gene sequence similarity of 95.73%) (Fig. 4). Considering the characteristics of *Streptomyces* sp. and the collected data of related *Streptomyces* species. It was determined that this strain could be identified as *Streptomyces griseorubens* strain NEAE-5.

### Placket-Burman design screening of significant variables

The Placket-Burman design was applied in order to screen and determine the significant process parameters affecting uricase production *Streptomyces griseorubens*. Eighteen assigned factors including L-asparagine, glutamic acid, allantoin, hypoxanthine, pH, incubation time, temperature, inoculum size, glycerol, glucose, yeast extract, KNO_3_, K_2_HPO_4_ NaCl, MgSO_4_.7H_2_O, and CaCl_2_, uric acid and peptone were chosen for the screening experiment, and their levels are presented in Table 3. The design matrix in Table 4 was used to screen these variables and evaluate their impact on uricase production.

**Table 3:** Experimental independent factors used for uricase production by *Streptomyces griseorubens* using Plackett–Burman design

Code	Variables (g/L)	Levels	
		− 1	+ 1
A	Uric acid	3	7
B	Glycerol	5	30
C	Glucose	0	10
D	Peptone	0.0	4
E	L-asparagine	0.0	4
F	Glutamic acid	0.0	4
G	Allantoin	0.0	4
H	Hypoxanthine	0.0	4
J	MgSO_4_.7H_2_O	0.1	0.5
K	KNO_3_	0.5	2
L	K_2_HPO_4_	0.5	2
M	NaCl	0.1	0.5
N	Yeast extract	0.0	0.5
O	CaCl_2_	0.1	0.5
P	Incubation time (days)	4	7
Q	pH	6	7.5
R	Temperature (^o^C)	28	37
S	Inoculum size (mL/100 mL medium)	2	4

**Table 4:** Twenty-trial Plackett–Burman experimental design along with experimental, predicted uricase production and residuals

Std	Run no	Coded levels of independent variables																			Uricase activity (U/mL)		Residuals
		A	B	C	D	E	F	G	H	J	K	L	M	N	O	P	Q	R	S	Dummy 1	Actual value	Predicted value	
15	1	1	1	1	− 1	1	− 1	1	− 1	− 1	− 1	− 1	1	1	− 1	1	1	− 1	− 1	1	57.74	58.03	− 0.29
10	2	− 1	1	− 1	− 1	− 1	− 1	1	1	− 1	1	1	− 1	− 1	1	1	1	1	− 1	1	70.74	70.94	− 0.20
4	3	1	1	− 1	1	1	− 1	− 1	1	1	1	1	− 1	1	− 1	1	− 1	− 1	− 1	− 1	67.56	67.27	0.29
12	4	− 1	1	− 1	1	− 1	− 1	− 1	− 1	1	1	− 1	1	1	− 1	− 1	1	1	1	1	20.37	20.57	− 0.20
2	5	− 1	1	1	− 1	− 1	1	1	1	1	− 1	1	− 1	1	− 1	− 1	− 1	− 1	1	1	19.16	19.45	− 0.29
11	6	1	− 1	1	− 1	− 1	− 1	− 1	1	1	− 1	1	1	− 1	− 1	1	1	1	1	− 1	49.36	49.16	0.20
13	7	1	− 1	1	− 1	1	− 1	− 1	− 1	− 1	1	1	− 1	1	1	− 1	− 1	1	1	1	68.56	68.85	− 0.29
17	8	− 1	1	1	1	1	− 1	1	− 1	1	− 1	− 1	− 1	− 1	1	1	− 1	1	1	− 1	11.31	11.11	0.20
18	9	− 1	− 1	1	1	1	1	− 1	1	− 1	1	− 1	− 1	− 1	− 1	1	1	− 1	1	1	52.46	52.74	− 0.29
6	10	− 1	− 1	1	1	− 1	1	1	− 1	− 1	1	1	1	1	− 1	1	− 1	1	− 1	− 1	33.53	33.33	0.20
5	11	− 1	1	1	− 1	1	1	− 1	− 1	1	1	1	1	− 1	1	− 1	1	− 1	− 1	− 1	18.33	18.13	0.20
7	12	− 1	− 1	− 1	1	1	− 1	1	1	− 1	− 1	1	1	1	1	− 1	1	− 1	1	− 1	43.70	43.42	0.29
14	13	1	1	− 1	1	− 1	1	− 1	− 1	− 1	− 1	1	1	− 1	1	1	− 1	− 1	1	1	30.18	30.38	− 0.20
9	14	1	− 1	− 1	− 1	− 1	1	1	− 1	1	1	− 1	− 1	1	1	1	1	− 1	1	− 1	57.44	57.15	0.29
19	15	1	− 1	− 1	1	1	1	1	− 1	1	− 1	1	− 1	− 1	− 1	− 1	1	1	− 1	1	39.26	39.46	− 0.20
16	16	1	1	1	1	− 1	1	− 1	1	− 1	− 1	− 1	− 1	1	1	− 1	1	1	− 1	− 1	97.22	97.03	0.20
3	17	1	− 1	1	1	− 1	− 1	1	1	1	1	− 1	1	− 1	1	− 1	− 1	− 1	− 1	1	55.37	55.66	− 0.29
8	18	− 1	− 1	− 1	− 1	1	1	− 1	1	1	− 1	− 1	1	1	1	1	− 1	1	− 1	1	47.21	47.41	− 0.20
1	19	1	1	− 1	− 1	1	1	1	1	− 1	1	− 1	1	− 1	− 1	− 1	− 1	1	1	− 1	36.25	35.96	0.29
20	20	− 1	− 1	− 1	− 1	− 1	− 1	− 1	− 1	− 1	− 1	− 1	− 1	− 1	− 1	− 1	− 1	− 1	− 1	− 1	51.23	50.94	0.29

The observed uricase production varied significantly across the twenty Plackett–Burman trials, ranging from 11.31 to 97.22 U/mL (Table 4), underscoring the need for medium optimization to achieve higher uricase yields. The wide disparities in production were attributed to the presence of diverse medium components at high and low levels. Multiple-regression analysis (Table 5) was performed to determine how independent variables affect uricase production.

**Table 5:** Regression statistics and ANOVA for the Plackett–Burman design experimental results

Source	Coefficient	Contribution %	Sum of squares	*Df*	Mean square	*F-*value	*P-*value
Intercept	46.35		8542.49	17	502.50	812.97	0.0012*
A-Uric acid	9.54	21.33	1822.03	1	1822.03	2947.79	0.0003*
B-Glycerol	− 3.46	2.81	239.86	1	239.86	388.05	0.0026*
C- Glucose	0.0	0.0005	−	1	−	−	−
D-Peptone	− 1.25	0.37	31.44	1	31.44	50.86	0.0191*
E-L-asparagine	− 2.11	1.04	89.14	1	89.14	144.22	0.0069*
F-Glutamic acid	− 3.25	2.47	210.66	1	210.66	340.82	0.0029*
G-Allantoin	− 3.9	3.56	304.07	1	304.07	491.94	0.002*
H-Hypoxanthine	7.55	13.36	1141.51	1	1141.51	1846.80	0.0005*
J- MgSO_4_.7H_2_O	− 7.81	14.29	1220.61	1	1220.61	1974.78	0.0005*
K-KNO_3_	1.71	0.69	58.56	1	58.56	94.75	0.0104*
L-K_2_HPO_4_	− 2.31	1.25	106.83	1	106.83	172.84	0.0057*
M-NaCl	− 7.14	11.95	1020.65	1	1020.65	1651.26	0.0006*
N- Yeast extract	4.9	5.62	480.22	1	480.22	776.93	0.0013*
O-CaCl_2_	3.66	3.13	267.55	1	267.55	432.86	0.0023*
P-Incubation time	1.4	0.46	39.36	1	39.36	63.67	0.0153*
Q-pH	4.31	4.36	372.21	1	372.21	602.18	0.0017*
R-Temperature (^o^C)	1.03	0.25	21.32	1	21.32	34.49	0.0278*
S-Inoculum size	− 7.47	13.07	1116.47	1	1116.47	1806.28	0.0006*
Std. Dev	0.79	R^2^	0.9999				
Mean	46.35	Adj R^2^	0.9986				
C.V. %	1.70	Pred R^2^	0.9855				
PRESS	123.62	Adeq Precision	115.20				

“* Significant values, *df*: Degree of freedom, *F*: Fishers’s function, *P*: Level of significance, C.V: Coefficient of variation, PRESS: predicted residual sum of squares”

**Fig. 5:**
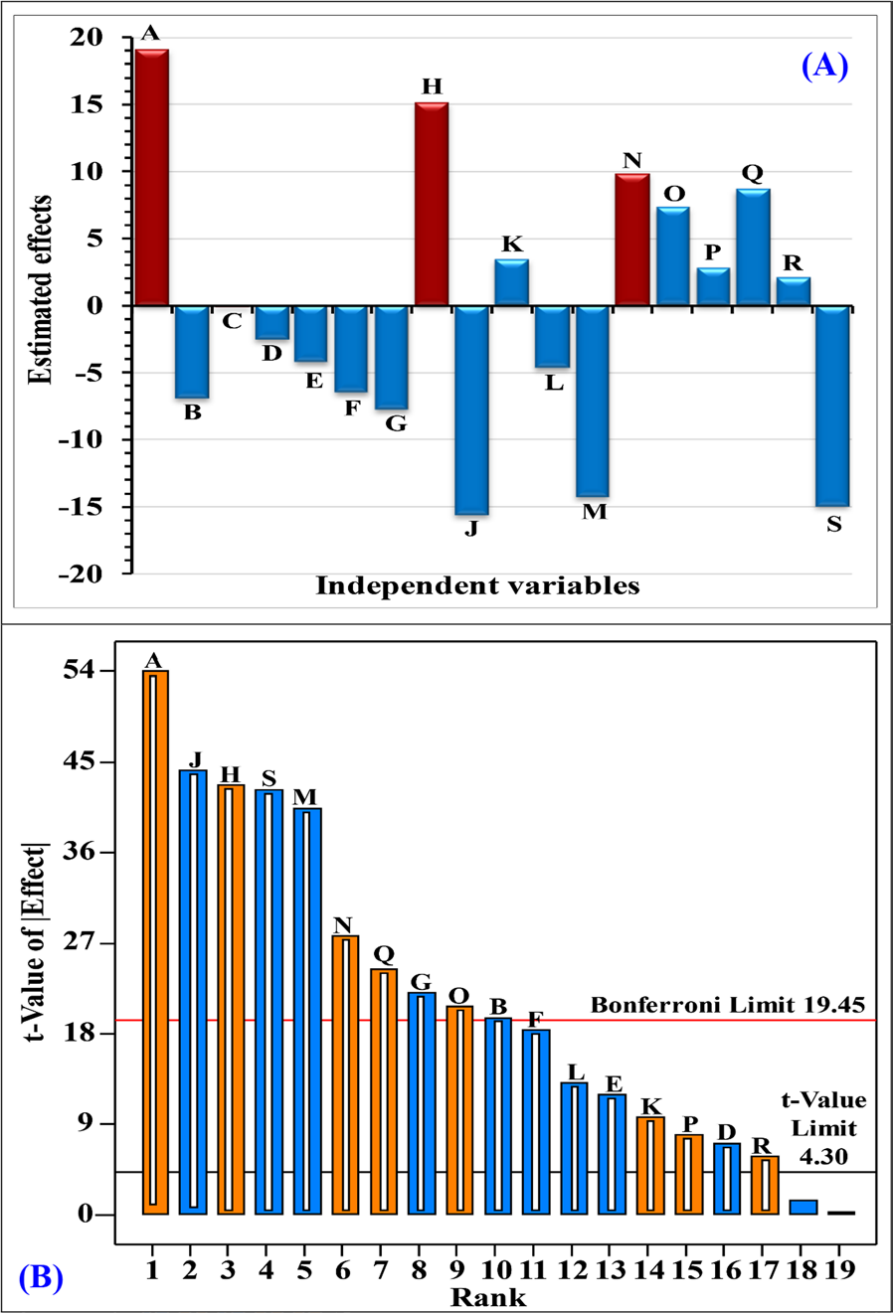
**A** Estimated effects plot determined using Plackett–Burman design **B** Pareto chart demonstrates the relationship between ranks and* t*-value

The main effects of different parameters were calculated based on the difference in averages between low (-1) and high (+ 1) levels. Among the variables that were studied; uric acid, hypoxanthine, KNO_3_, yeast extract, CaCl_2_, incubation time, pH and temperature had positive effects on production of uricase. Whereas, glycerol, glucose, peptone, L-asparagine, glutamic acid, allantoin, MgSO_4_.7H_2_O, K_2_HPO_4_, NaCl, and inoculum size had negative effects on production of uricase (Table 5, Fig. 5A). The magnitude of the estimated effects, whether positive or negative, indicated the significant influence of these variables on uricase production. Conversely, a nearly null estimated effect suggests the variable has little to no impact on uricase production [43]. Table 5 displays the contributions percentages of the variables. The percentage contribution can be used to determine the extent to which each factor contributed to the variation in the response. Moreover, the results revealed that the uric acid, hypoxanthine, MgSO_4_.7H_2_O, NaCl, yeast extract, pH, and inoculum size have the most the contributions, with percentages of 21.33, 13.36, 14.29, 11.95, 5.62, 4.36 and 13.07, respectively.

The process factors affecting uricase production are presented in the order of their significance using a Pareto chart (Fig. 5B), along with the corresponding effect values of various medium components and environmental conditions. The Pareto chart presents the absolute values of the factors effect and includes a reference line. Effects exceeding the reference line are considered significant. Additionally, the Pareto chart demonstrates the relationship between ranks and* t*-value (effect). Based on the Pareto chart (Fig. 5B), uric acid (A) exhibited the highest effect on uricase production, followed by MgSO_4_.7H_2_O (J), hypoxanthine (H), inoculum size (S), NaCl (M), yeast extract (N) and pH (Q) with effect values of 19.08, -15.62, 15.1, -14.94, -14.28, 9.8 and 8.62; respectively.

Analysis of variance (ANOVA) was performed on the data obtained from the uricase production experiments, and the corresponding findings are presented in Table 5. The coefficients and model significance were determined using the *P*-values and *F*-values. Lower *P*-values suggest greater significance of the factors influencing uricase production. Some researchers consider confidence levels above 70% as acceptable [29] However, El-Naggar et al*.* [44] stated that the significant variables were those with confidence intervals higher than 90% (*P* < 0.1). When the *P*-value of a model term is less than 0.05, it is considered to be statistically significant [45]. In this study, factors were considered to significantly affect uricase production if their *P*-values were less than 0.05. The model's *P*-value (0.0012) and *F*-value (812.97) indicate its overall significance. Among the factors analyzed, all except glucose affected uricase production significantly with a *P-*values were below 0.05 (Table 5). The significant variables including uric acid, hypoxanthine, KNO_3_, yeast extract, CaCl_2_, incubation time, pH and temperature, had positive impacts on uricase production by *Streptomyces griseorubens*. On the contrary, glycerol, glucose, peptone, L-asparagine, glutamic acid, allantoin, MgSO_4_.7H_2_O, K_2_HPO_4_, NaCl, and inoculum size had negative impacts on uricase production by *Streptomyces griseorubens*.

The model's adequacy was evaluated using the determination coefficient (R^2^). R^2^ measures the proportion of variability in the experimental results that can be explained by the selected factors [27]. R^2^ values are always between zero and one. When the values of R^2^ are near to one, the model is robust and predicts the response more accurately [46]. R^2^ values above 0.9 were considered statistically significant and indicates that the predicted and experimental response values are highly correlated [43]. The current value of R^2^ for statistical analysis of the experimental results is 0.9999, indicating that the selected independent factors can explain 99.99% of the observed variation in uricase production. Only 0.1% of the variability remains unexplained by the chosen independent factors. This high R^2^ value indicates that the predicted and experimental uricase production values are highly correlated, confirming the reliability of the utilized model for uricase production. Furthermore, the model is reliable, as indicated by the adjusted R^2^ value of 0.9986 [36]. The model's ability to predict future uricase production is supported by the high predicted R^2^ value of 0.9855. The adequate precision value is 115.20. A signal-to-noise ratio that is more than four is considered to be ideal [47]. The coefficient of variation percentage (C.V. %) value of 1.70% suggests a greater level of model precision. In general, a lower C.V. % indicates a higher degree of precision of the experimental model. The small value of PRESS (123.62) shows a higher accuracy and precision. The standard deviation of the model is 0.79, while the mean value is 46.35.

### Model adequacy checking

**Fig. 6:**
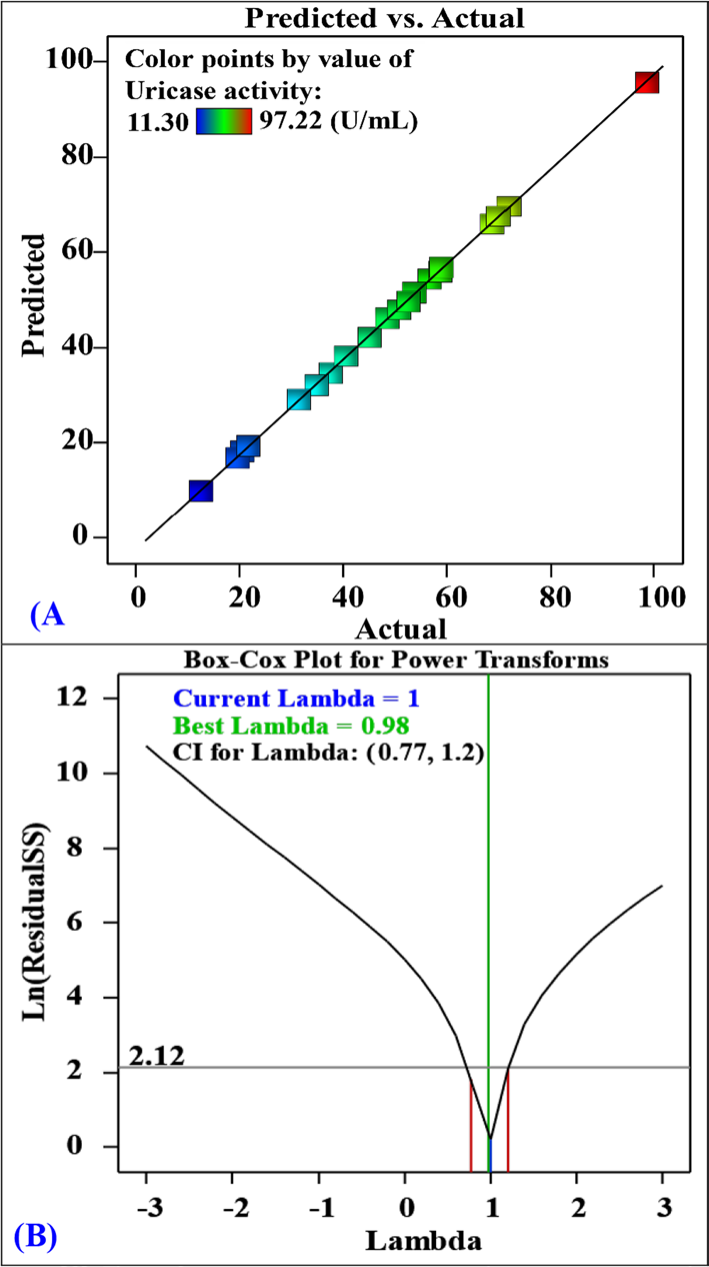
**A** plot of predicted versus actual uricase production and **B** Box-Cox plot of model transformation

Model adequacy was assessed through various analyses. Figure 6A depicts a plot of the predicted uricase production values versus the experimental (actual) uricase production values. The tightness of the data points around the regression line provides evidence of a significant correlation between the predicted values by the model and the experimental data of uricase production, thereby validating the accuracy of the model [44]. Furthermore, Fig. 6B presents a Box-Cox plot of model transformation. The optimal Lambda (λ) value of 0.98, green line, lies between the two vertical red lines, which indicate the low and high 95% confidence levels (0.77 and 1.2, respectively). This finding shows that there is no need for additional data transformation and the model is appropriate.

The following first-order polynomial equation was obtained (by ignoring the non-significant factors) and can be used to predict uricase production by *Streptomyces griseorubens* for specific levels of each variable:

3 $$Uricase activity (U/mL) = +9.54\times A-3.46\times B-1.25\times D-2.11\times E-3.25\times F-3.9\times G+7.55\times H-7.81\times J +1.71\times K-2.31\times L 7.14\times M+4.9\times N+3.66\times O+1.4\times P+4.31\times Q+1.03\times R-7.47\times S$$

where A, B, D, E, F, G, H, J, K, L, M, N, O, P, Q, R and S are uric acid, glycerol, peptone, L-asparagine, glutamic acid, allantoin, hypoxanthine, yeast extract, KNO_3_, K_2_HPO_4_, NaCl, MgSO_4_.7H_2_O, CaCl_2_, incubation time, pH, temperature, and inoculum size; respectively.

Equation 3 showed that the coefficients of uric acid, hypoxanthine, KNO_3_, yeast extract, CaCl_2_, incubation time, pH and temperature were positive; while the coefficients of glycerol, glucose, peptone, L-asparagine, glutamic acid, allantoin, MgSO_4_.7H_2_O, K_2_HPO_4_, NaCl, and inoculum size were negative. So, the increase in uric acid, hypoxanthine, KNO_3_, yeast extract, CaCl_2_, incubation time, pH and temperature levels and decrease in the levels of glycerol, glucose, peptone, L-asparagine, glutamic acid, allantoin, MgSO_4_.7H_2_O, K_2_HPO_4_, NaCl, and inoculum size improve uricase production by *Streptomyces griseorubens*.

An experiment was conducted to verify the Plackett–Burman design's accuracy using a medium composed of the following quantities (in g/L): uric acid 7, glycerol 5, hypoxanthine 4, MgSO_4_.7H_2_O 0.1, KNO_3_ 2, CaCl_2_ 0.5, K_2_HPO_4_ 0.5, NaCl 0.5, yeast extract 0.5. In addition, the period of incubation is seven days, pH of 7.5, temperature of 37 °C, and an inoculum size of 2 mL/100 mL medium. The resulting uricase activity was measured as 90.75 U/mL, which is approximately three times higher than the uricase production achieved using the basal medium prior to implementing the Plackett–Burman design (30.45 U/mL).

### CCD-based optimization of key factors

Plackett–Burman results analysis revealed that uric acid, hypoxanthine, and yeast extract concentrations were the most significant factors positively affecting uricase production and had the highest contribution. Therefore, the CCD was used to further optimize the three factors and to improve uricase production. Table 6 shows the coded and actual levels of uric acid, hypoxanthine, and yeast extract concentrations selected for CCD-based optimization. CCD matrix, experimental and predicted uricase activities for the 20 experiments used are displayed in Table 6. The obtained uricase activity ranged from 29.45 to 126.67 U/mL, with the highest level observed in run number 5, where the uric acid, hypoxanthine, and yeast extract concentrations were 7, 5, and 1 g/L, respectively. The predicted uricase production values determined by the model and the experimentally measured values are presented in Table 6.

**Table 6:** Central composite design representing coded and actual levels of the process variables, the experimental, predicted uricase production and residuals

Std	Run	Type	Variables			Uricase activity (U/mL)		Residuals
			X_1_	X_2_	X_3_	Experimental	Predicted	
11	1	Axial	0	− 1.68	0	55.93	54.79	1.14
3	2	Factorial	− 1	1	− 1	44.00	44.03	− 0.03
18	3	Center	0	0	0	121.86	124.16	− 2.31
4	4	Factorial	1	1	− 1	56.85	55.06	1.80
17	5	Center	0	0	0	126.67	124.16	2.51
19	6	Center	0	0	0	121.67	124.16	− 2.49
6	7	Factorial	1	− 1	1	29.45	28.46	0.98
7	8	Factorial	− 1	1	1	94.85	93.19	1.66
12	9	Axial	0	1.68	0	79.26	81.74	− 2.48
8	10	Factorial	1	1	1	96.52	95.01	1.51
14	11	Axial	0	0	1.68	48.33	49.84	− 1.51
16	12	Center	0	0	0	124.26	124.16	0.10
15	13	Center	0	0	0	125.00	124.16	0.84
2	14	Factorial	1	− 1	− 1	60.37	61.09	− 0.72
5	15	Factorial	− 1	− 1	1	54.26	55.11	− 0.85
13	16	Axial	0	0	− 1.68	36.11	35.94	0.17
20	17	Center	0	0	0	125.74	124.16	1.58
9	18	Axial	− 1.68	0	0	97.52	97.19	0.33
10	19	Axial	1.68	0	0	82.39	84.06	− 1.67
1	20	Factorial	− 1	− 1	− 1	77.96	78.53	− 0.56

Level	Uric acid conc. (g/L)	Hypoxanthine conc. (g/L)	Yeast extract conc. (g/L)
− 1.68	1.95	1.64	0.16
− 1	4	3	0.5
0	7	5	1
1	10	7	1.5
1.68	12.05	8.36	1.84

### Multiple regression analysis and ANOVA

Multiple regression analysis was performed on the dataset generated from the CCD experiments, and the resulting findings are shown in Tables 7, 8. The adequacy of the model is confirmed by the R^2^ value, which is determined to be 0.9979 (Table 7) highlights the model's accuracy. Additionally, the adjusted R^2^ value of 0.996 further validates the model significance. The high predicted R^2^ value of 0.9901 indicates a significant proximity between the predicted and experimental values of uricase production. Fisher's *F*-test value of 531.61 and very small *P*-value (< 0.0001) (Table 7) suggest that the model is highly significant. *F* and *P-*values reveals that the concentrations of uric acid, yeast extract, and hypoxanthine exert significant effects, as indicated by the significance of their linear coefficients, interaction effects, and quadratic effects. This suggests that these three variables are limiting factors, and even slight variations in their levels can impact the production of uricase by *Streptomyces griseorubens*. The interaction between two variables can be expressed as a positive coefficient (a synergistic effect) or a negative coefficient (an antagonistic effect) (synergistic effect) [48]. The fact that the coefficients for X_2_, X_3_, X_1_X_2_, and X_2_X_3_ are all positive shows that the linear effect of X_2_, X_3_, as well as the interaction effects of X_1_, X_2_, and X_2_X_3_ all contribute to an increase in uricase production, whereas negative coefficients show a decline in uricase production. The "Adeq Precision" ratio is 64.17, while the coefficient of variance (C.V.) value of 2.54 indicates the accuracy of the experimental results. Furthermore, the PRESS value of 211.21, standard deviation value of 2.11, and mean value of 82.95 (Table 7) provide additional measures of the model's performance.

**Table 7:** ANOVA for the design of CCD used for the production of uricase by *Streptomyces griseorubens*

Source of variance		Sum of Squares	*df*	Mean Square	*F-*value	*P-*value *P*rob > *F*	Coefficient estimate
Model		21,279.49	9	2364.39	531.61	< 0.0001*	124.16
Linear effects	X_1_	208.25	1	208.25	46.82	< 0.0001*	− 3.90
	X_2_	876.75	1	876.75	197.13	< 0.0001*	8.01
	X_3_	233.21	1	233.21	52.43	< 0.0001*	4.13
Interaction effects	X_1_ X_2_	405.17	1	405.17	91.10	< 0.0001*	7.12
	X_1_ X_3_	42.34	1	42.34	9.52	0.0115*	− 2.30
	X_2_ X_3_	2633.52	1	2633.52	592.12	< 0.0001*	18.14
Quadratic effects	X_1_^2^	2025.97	1	2025.97	455.52	< 0.0001*	− 11.86
	X_2_^2^	5628.60	1	5628.60	1265.54	< 0.0001*	− 19.76
	X_3_^2^	11,898.24	1	11,898.24	2675.20	< 0.0001*	− 28.73
Error effects	Lack of Fit	23.44	5	4.69	1.11	0.4541	124.16
	Pure Error	21.03	5	4.21			− 3.90

Std. Dev	2.11	R^2^	0.9979
Mean	82.95	Adj R^2^	0.996
C.V. %	2.54	Pred R^2^	0.9901
PRESS	211.21	Adeq Precision	64.17

“* Significant values,* P*: Level of significance,* F*: Fishers’s function, *df*: Degree of freedom,”

**Table 8:** Fit summary of CCD used for the production of uricase by *Streptomyces griseorubens*

Sequential model sum of squares					
Source	Sum of Squares	*df*	Mean Square	*F-*value	*P-*value
Linear vs Mean	1318.21	3	439.40	0.35	0.7887
2FI vs Linear	3081.03	3	1027.01	0.79	0.5214
Quadratic vs 2FI	16,880.25	3	5626.75	1265.12	< 0.0001*

Fit summary				
Source	Sequential *P*-value	Lack of Fit *P*-value	Adjusted R^2^	Predicted R^2^
Linear	0.7887	< 0.0001*	− 0.1141	− 0.3796
2FI	0.5214	< 0.0001*	− 0.16	− 0.9015
Quadratic	< 0.0001	0.4541	0.996	0.9901

Lack of fit tests					
Source	Sum of Squares	*df*	Mean Square	*F-*value	*P-*value
Linear	19,984.72	11	1816.79	431.86	< 0.0001*
2FI	16,903.69	8	2112.96	502.26	< 0.0001*
Quadratic	23.44	5	4.69	1.11	0.4541

Model summary statistics					
Source	Standard deviation	R-Squared	Adjusted R-Squared	Predicted R-Squared	PRESS
Linear	35.36	0.0618	− 0.1141	− 0.3796	29,417.7
2FI	36.08	0.2063	− 0.16	− 0.9015	40,548.6
Quadratic	2.11	0.9979	0.996	0.9901	211.21

“Two factors interaction: 2FI, PRESS: sum of squares of prediction error

*Significant values, *df*: degree of freedom”

The results of the fit summary presented in Table 8 determine the adequacy of the quadratic model for uricase production by *Streptomyces griseorubens*. Quadratic model is selected as it exhibits great significance, with an extremely low *P-* value of < 0.0001, an insignificant lack of fit (*P*-value = 0.4541), a higher adjusted R^2^ (0.996) and predicted R^2^ (0.9901), and the lowest standard deviation of 2.11. Uricase production by *Streptomyces griseorubens* can be predicted using coefficients for independent process variables by the equation of second order polynomial:

4 $${\text{Uricase activity}} = + {124}.{16 }{-}{3}.{9}0{\text{X}}_{{1}} + {8}.0{\text{1X}}_{{2}} + {4}.{\text{13X}}_{{3}} + {7}.{\text{12X}}_{{1}} {\text{X}}_{{2}} {-}{2}.{3}0{\text{X}}_{{1}} {\text{X}}_{{3}} + {18}.{\text{14X}}_{{2}} {\text{X}}_{{3}} {-}{11}.{\text{86X}}_{{1}}^{{2}} {-}{19}.{\text{76X}}_{{2}}^{{2}} {-}{28}.{\text{73X}}_{{3}}^{{2}}$$

where, X_1_, X_2_ and X_3_ are uric acid, hypoxanthine and yeast extract concentrations; respectively.

### Three dimensional (3D) plots

**Fig. 7:**
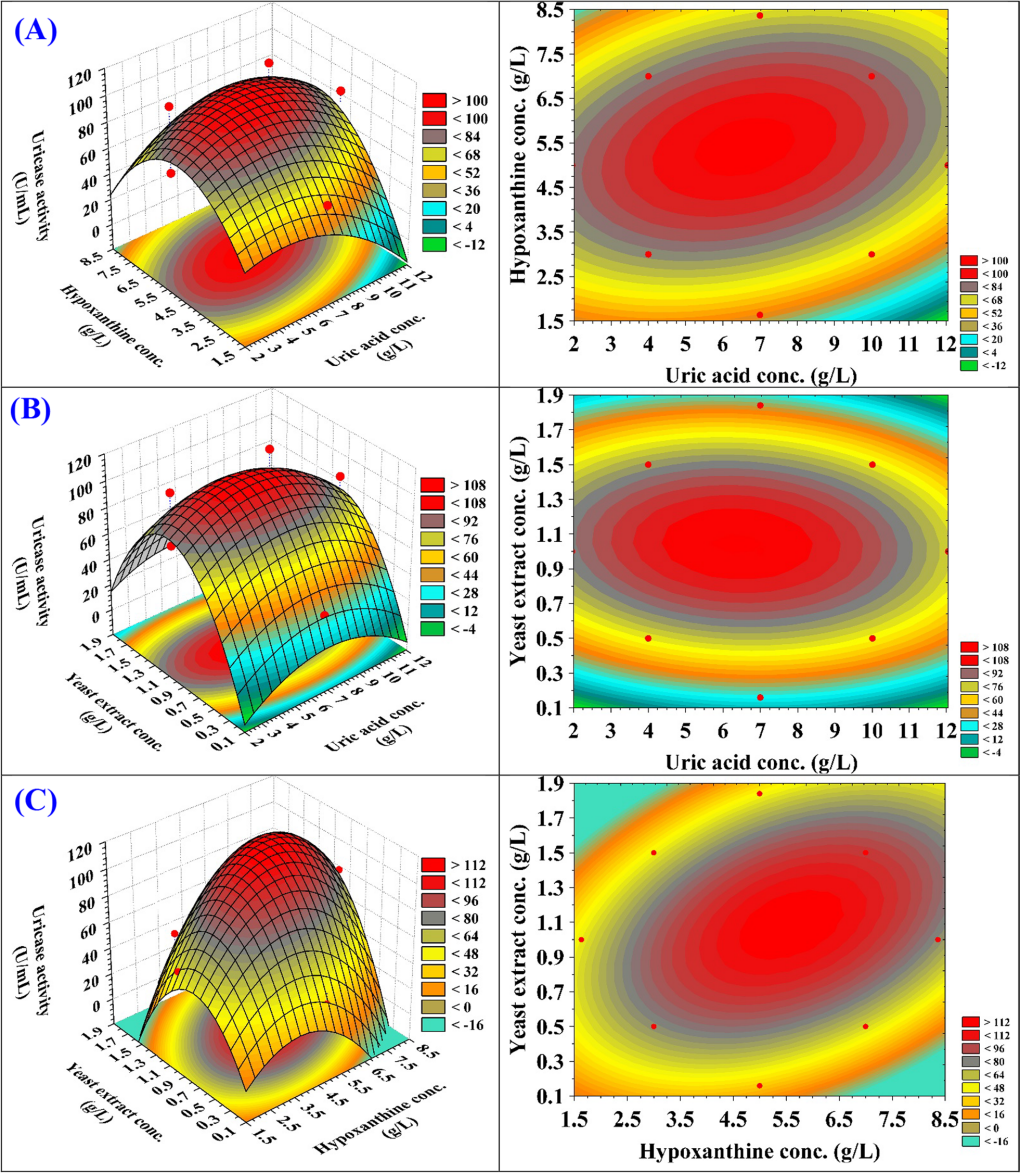
**A-C** 3D response surface and 2D Contour plots showing the interaction effects of uric acid concentration (X_1_), hypoxanthine concentration (X_2_) and yeast extract concentration (X_3_) on uricase production by *Streptomyces griseorubens*. Contour plots are on the right, whereas 3D surface plots are on the left

Three-dimensional (3D) and contour plots can be used to evaluate the correlation between uricase production by *Streptomyces griseorubens* and the interactions among test factors in order to determine the best possible conditions, as shown in Fig. 7. The 3D and contour plots in Fig. 7A show the impact of uric acid concentration and hypoxanthine concentration on uricase production, with the concentration of yeast extract held at zero. The uricase production increases gradually with increasing both uric acid and hypoxanthine concentrations. However, beyond 6.6 g/L of uric acid concentration and 5.5 g/L of hypoxanthine concentration, exhibits a negative effect and resulting in a decline in uricase activity.

Uric acid was employed as a nitrogen source in the basic media to induce uricase production by *Streptomyces griseorubens*. *Streptomyces griseorubens* uricase activity under the conditions of submerged fermentation was determined to be 65.51 U/mL with a uric acid concentration of 5 g/L, uric acid concentration greater than 5 g/L did not increase uricase production. These results agreed with El-Naggar [31] who indicated that, the uricase production was inhibited by high concentration of uric acid. Yazdi et al. [22] stated that, maximum uricase production is induced at a concentration of 7 g/L by the uric acid-containing medium. Also, Lotfy [49] demonstrated that highest level of uricase activity was seen when uric acid was present, indicating that uricase is an inducible enzyme. Overall, the utilisation of organic nitrogen resulted in an enhancement of uricase production. Organic nitrogen has the most diverse array of amino acids, which are crucial for microbial growth, are easily assimilated by cells, and induce uricase production.

The maximal value of uricase activity produced by *Aspergillus niger* was documented by Abbas [50] when the sole nitrogen source in the fermentation medium was uric acid at a concentration of 0.5 percent. The study conducted by Geweely and Nawar [23] demonstrated that the activities of extracellular and intracellular uricase were at their maximum at a uric acid concentration of 0.1 percent. Furthermore, on the 5th day of incubation, uricase activity of *Gliocladium virde* in the uric acid medium was 63.14 U/mL when the only nitrogen source used was uric acid at a concentration of 3 g/L [14]. Also, Jianguo et al. [24] showed that the maximum uricase production was obtained by* Candida utilis* in the fermentation medium that contain 6 g/L uric acid as the source of nitrogen. Ram et al. [51] revealed that the maximal uricase production was 73.61U/L when the medium contained 3 g/L of uric acid; however, uric acid concentrations beyond 0.3% failed to increase uricase production. The production of uricase was inhibited by a high uric acid concentration. Ghasemian et al. [52] demonstrated that the optimal concentration of uric acid for uricase production has been determined to be 0.4 percent. The most effective inducer for uricase production (19.41 U/mL) by *Bacillus cereus* was uric acid (19.41 U/mL) [53]. Uricase production increased with rising uric acid concentrations until it reached 2 g/L, after which it began to decline. This could be because the enzyme inhibited the substrate.

Figure 7B represents the interaction between the concentrations of uric acid and yeast extract while the hypoxanthine concentration is maintained at zero level. By increasing both uric acid concentration and yeast extract concentration, uricase production increases gradually and the maximal uricase production was achieved at 6.6 g/L of uric acid and 1.08 g/L of yeast extract, and any further increase in uric acid or yeast extract leads to a decrease in uricase production. Also, Fig. 7B showed that low and high levels of uric acid concentration led to low levels of uricase production by *Streptomyces griseorubens*.

The use of organic nitrogen sources led to a significantly greater increase in uricase production in comparison to inorganic nitrogen sources. It is hypothesized that organic nitrogen sources are more complicated in nature, provide a greater abundance of amino acids that are essential for microbial growth and are readily absorbed by the cells to stimulate uricase production [4]. *Aspergillus welwitschiae* was able to produce the highest uricase when the yeast extract concentration was set to 2 g/L, whereas increased or decreased concentrations of yeast extract lowered uricase production [4]. Yeast extracts contain nucleotides and polysaccharides particularly α-mannan and β-glucan, which promote uricase production. Anderson and Vijayakumar [54] found that *Pseudomonas aeruginosa* was able to produce uricase when using 2 g/L of yeast extract. Abbas [50] recorded the highest uricase activity by *Aspergillus niger* when the fermentation medium contained 2% yeast extract. Nanda et al*.* [14] observed that *Gliocladium viride* MTCC 3835 produce the maximum value of uricase activity (82.1 U/mL) using 10.57 g/L yeast extract. Whereas, *Pseudomonas* sp. produce the maximum value of uricase activity (0.23 U/mL) using 0.5% yeast extract concentration [52]. Khucharoenphaisan and Sinma [55] found that *Saccharopolyspora* sp. PNR11 generated 216 mU/mL of uricase when yeast extract was used as a nitrogen source at a concentration of 1%.

The interaction between the concentrations of hypoxanthine and yeast extract is shown in Fig. 7C, with the uric acid concentration being held constant at zero level. Maximum uricase production was obtained when yeast extract concentration was around 1.08 g/L. Uricase production increased alongside an increase in yeast extract concentration. Further elevation of yeast extract concentration leads to a decrease in uricase production.

### Model adequacy checking

**Fig. 8:**
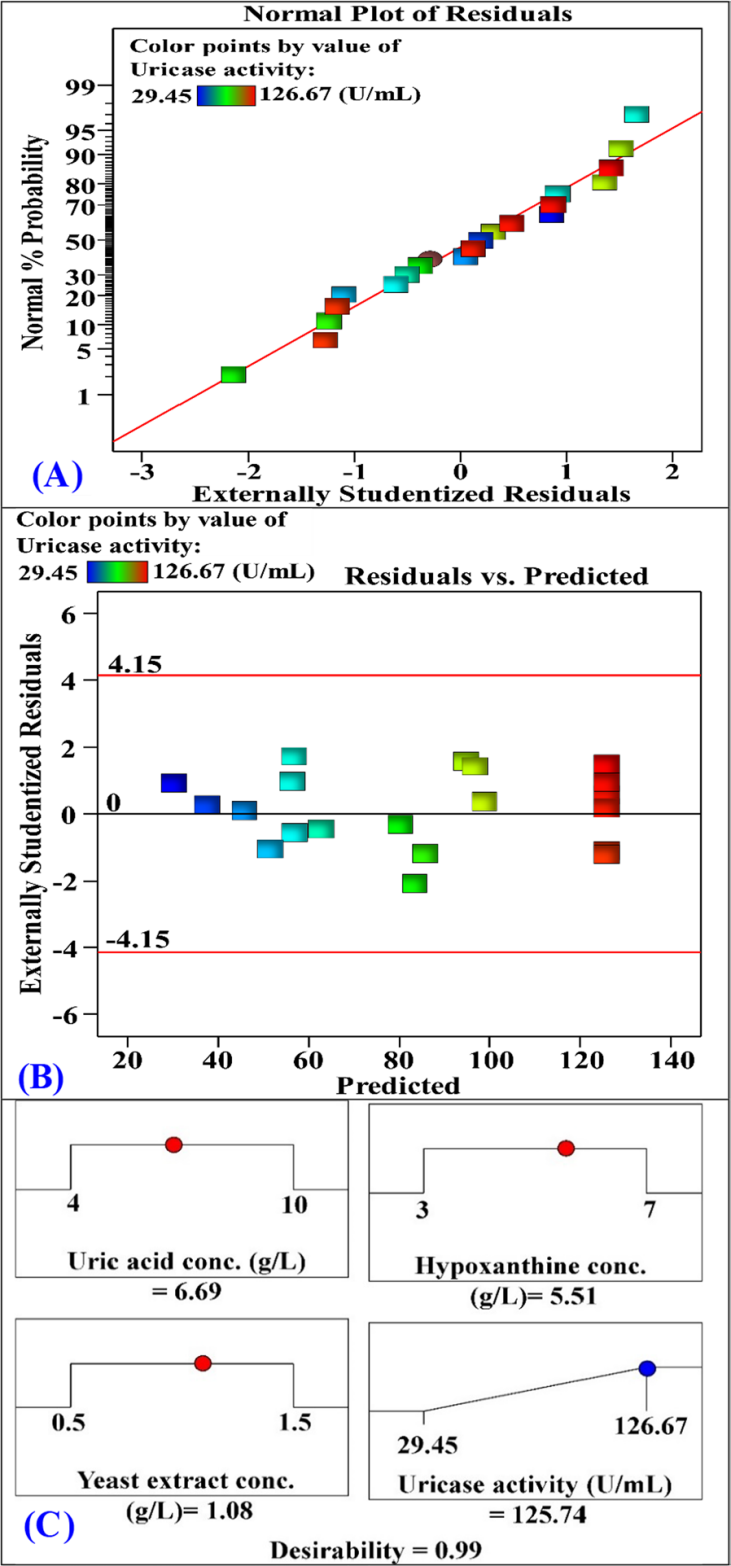
**A** Normal probability plot of residuals, and **B** plot of internally studentized residuals versus predicted uricase production, and **C** the optimum predicted values for maximum uricase production by *Streptomyces griseorubens* and the desirability value

The normal probability plot (NPP) was employed to evaluate whether the residuals conform to a normal distribution to validate the model suitability. The differences between the experimental and theoretical values of the responses are known as the residuals. Minimal residual values indicate that the model's prediction is highly precis [56]. Figure 8A displays the normal probability plot. The residuals follow a nearly straight line, suggesting a close match between the predicted and observed uricase production. Furthermore, Fig. 8B illustrates the residuals plot against the predicted uricase production. The plot demonstrates a random scattering of residuals, indicating that the residuals are distributed randomly. This further confirms the adequacy of the model in optimizing uricase production using the central composite design.

### Model verification

To validate the accuracy of the model, a desirability function was employed to determine the theoretically optimal conditions for maximizing uricase production (125.74 U/mL). Figure 8C presents the predicted optimal conditions for maximizing uricase production, which include uric acid (6.96 g/L), hypoxanthine (5.51 g/L), and yeast extract (1.08 g/L). Subsequently, an experiment was carried out using the conditions that were predicted to produce a maximum uricase activity. The experimental result obtained under the predicted optimal conditions showed a maximum uricase activity of 120.35 U/mL. The experimental result closely approximates the predicted value of 125.74 U/mL, demonstrating that the model's predictions are very accurate and reliable. The statistical modeling is a robust model towards optimizing of bioprocesses.

## Data Availability

All data generated or analyzed during this study are included in this article.
